# St. Mary's Hospital Extension

**Published:** 1902-04-19

**Authors:** 


### ST. MARY'S HOSPITAL EXTENSION.

The new works have been commenced in connection with the extension, and are at present in active progress. It was a considerable feat to raise £50,000 to complete the Clarence Memorial Wing, and we are glad to notice that the energy and ability displayed in the matter by the Secretary, Mr. Thomas Ryan, have been suitably recognised by the Board on page 19 of the report, from which we reproduce the following paragraph:—

"By direction of the Board of Management, I hereby record its high sense of the eminent services rendered to the Hospital by the Secretary, Mr. Thomas Ryan, in connection with the raising of the £50,000 required to complete the Clarence Memorial Wing.

The ability with which Mr. Ryan stated the case for the Hospital to the Zunz executors, and conducted the negotiations with them, and the skill and perseverance displayed by him in raising the further £25,000 required, were conspicuous.

This is but one of many signal services rendered to the Hospital by Mr. Ryan in matters of exceptional importance or of special difficulty during his fifteen years of office, and the Board feel that he fully merits the distinction which it now has the pleasure to confer upon him, of special mention in the Annual Report.

Stanley G. Bird, *Chairman*."

*February 28th*, 1902.

We congratulate Mr. Thomas Ryan on the compliment which has been extended to him by the Committee of Management, which is well deserved, and should act as an encouragement to other zealous hospital secretaries throughout the country.

**"The Hospital" Institutional Library.**

"Hospitals and Asylums of the World." 4 Vols., with a Portfolio of Plans, complete £12 12s.

"Burdett's Hospitals and Charities, 1902." 5s.

"Cottage Hospitals, General, Fever, and Convalescent." 10s. 6d.

"Hospital Expenditure: The Commissariat." 2s. 6d.

"A Legal Handbook for the Use of Hospital Authorities." 2s. 6d.

"The Cottage Hospital Case Book or Register of Patients." 10s. and 12s. 6d.

"The Furnishing and Appliances of a Cottage Hospital." 6d.

All these are published by the Scientific Press, Ltd., and may be obtained through any bookseller, or direct from the publishers, 28 and 29 Southampton Street, Strand, London, W.C.

